# Bacterial Communities Associated with Host-Adapted Populations of Pea Aphids Revealed by Deep Sequencing of 16S Ribosomal DNA

**DOI:** 10.1371/journal.pone.0120664

**Published:** 2015-03-25

**Authors:** Jean-Pierre Gauthier, Yannick Outreman, Lucie Mieuzet, Jean-Christophe Simon

**Affiliations:** 1 INRA, UMR 1349 IGEPP "Institut de Génétique, Environnement et Protection des Plantes", 35653, Le Rheu, France; 2 Agrocampus Ouest, UMR 1349 IGEPP "Institut de Génétique, Environnement et Protection des Plantes", 35042, Rennes, France; Universite Pierre et Marie Curie, FRANCE

## Abstract

Associations between microbes and animals are ubiquitous and hosts may benefit from harbouring microbial communities through improved resource exploitation or resistance to environmental stress. The pea aphid, *Acyrthosiphon pisum*, is the host of heritable bacterial symbionts, including the obligate endosymbiont *Buchnera aphidicola* and several facultative symbionts. While obligate symbionts supply aphids with key nutrients, facultative symbionts influence their hosts in many ways such as protection against natural enemies, heat tolerance, color change and reproduction alteration. The pea aphid also encompasses multiple plant-specialized biotypes, each adapted to one or a few legume species. Facultative symbiont communities differ strongly between biotypes, although bacterial involvement in plant specialization is uncertain. Here, we analyse the diversity of bacterial communities associated with nine biotypes of the pea aphid complex using amplicon pyrosequencing of 16S rRNA genes. Combined clustering and phylogenetic analyses of 16S sequences allowed identifying 21 bacterial OTUs (Operational Taxonomic Unit). More than 98% of the sequencing reads were assigned to known pea aphid symbionts. The presence of *Wolbachia* was confirmed in *A*. *pisum* while *Erwinia* and *Pantoea*, two gut associates, were detected in multiple samples. The diversity of bacterial communities harboured by pea aphid biotypes was very low, ranging from 3 to 11 OTUs across samples. Bacterial communities differed more between than within biotypes but this difference did not correlate with the genetic divergence between biotypes. Altogether, these results confirm that the aphid microbiota is dominated by a few heritable symbionts and that plant specialization is an important structuring factor of bacterial communities associated with the pea aphid complex. However, since we examined the microbiota of aphid samples kept a few generations in controlled conditions, it may be that bacterial diversity was underestimated due to the possible loss of environmental or transient taxa.

## Introduction

Sustained associations between microbes and eukaryotic hosts are widespread. This is particularly true for insects which are engaged in multiple forms of interactions with micro-organisms referred to as symbiosis in its broad sense (i.e. all types of close associations between organisms of different species). Many arthropods have established intimate and mutualistic partnerships with obligate symbionts which generally supplement the host’s diet in key nutrients [1], [2]. In addition, researches over the last decades have highlighted the taxonomic diversity and the many roles of facultative symbionts. Although not essential for insect reproduction and survival, facultative symbionts may have indeed profound and sometimes spectacular effects on host ecology, physiology and behavior [3], [4]. However, until recently, the exploration of insect symbiont diversity was limited by the use of taxon-specific detection techniques or DNA-limiting, time consuming and low throughput methods. The development of Next Generation Sequencing techniques (NGS) now permits to assess without *a priori* the diversity and structure of insect microbial communities to better understand both the influence of microbes on their host populations and the dynamics of symbiotic associations. Although it is now possible to characterize and analyze the whole microbial community of a given host, these techniques have been mainly restricted so far to insect model species or species of medical importance [5], [6], [7], [8].

Aphids represent a well-studied case of symbiotic associations. In addition to their obligate symbiont (*Buchnera aphidicola*), these insects may harbour one or several heritable facultative bacterial symbionts. These symbionts have been mostly studied in the pea aphid, *Acyrthosiphon pisum*, a pest of legume crops. So far, eight bacterial facultative symbiont taxa of the pea aphid are known: the five Gammaproteobacteria *Hamiltonella defensa*, *Regiella insecticola*, *Serratia symbiotica* [9], *Rickettsiella viridis* [10, 11] and PAXS (Pea Aphid X-type Symbiont), [12]; two Alphaproteobacteria of the genera *Rickettsia* [13] and *Wolbachia* [14]; and finally *Spiroplasma* [15] belonging to Mollicutes. In pea aphids, facultative symbionts reside in different parts of the host including the hemolymph, sheath cells associated with the primary endosymbiont bacteriome or within bacteriocytes themselves [9]. These facultative symbionts may benefit their host through increased performance on specific plants [16], body colour change [11], heat tolerance [17] or protection against natural enemies [18], [19]. Although transmitted vertically with high fidelity, these symbionts may occasionally move horizontally within and between species [3], [20]. Besides heritable symbionts, little is known about gut associates, the bacteria colonizing the aphid digestive tract. More generally, we lack a deep characterization of the pea aphid microbiome and a better assessment of changes in bacterial communities according to environmental factors and host genotypes. Here, we used deep 454 amplicon pyrosequencing of 16S rDNA genes, to analyze the diversity and structure of bacterial communities associated with pea aphid populations specialized on nine different host plants.

The pea aphid consists of a series of host-adapted biotypes specialized on different host plants and showing a continuum of divergence from host races still exchanging some genes to genetically isolated incipient species. So far, 11 biotypes have been described, each adapted to one or a few legume species [21] and differing in their symbiotic complement [20], [22], [23].

Here, we examined the diversity of bacterial communities associated with nine biotypes of the pea aphid complex through combined clustering and phylogenetic analyses of 16S rDNA sequences. In particular, we were looking for unreported symbionts in the pea aphid and other bacterial taxa such as gut associates present in low abundance. We also assessed the importance of plant specialization on the structure of bacterial communities by comparing the microbiomes of the different pea aphid biotypes. Finally, we tested whether genetic divergence between aphid biotypes correlated with dissimilarity between their respective bacterial communities. In this study, we wanted to avoid the detection of microbes associated with internal parasites such as parasitic wasps (i.e. aphid hymenopteran parasitoids which are frequently encountered in field populations of aphids as “mummies”) and to focus our study on bacteria with prolonged associations with their hosts. Therefore, we analyzed the microbiota of aphid samples that were kept a few generations in controlled conditions, being however aware this could alter microbial diversity associated with pea aphids in the field.

## Material and Methods

### Pea aphid samples

Aphids were sampled from nine different plant species on which *A*. *pisum* is known to occur as host-specialized populations or biotypes [21]: *Cytisus scoparius* (common broom), *Lathyrus pratensis* (meadow vetchling), *Medicago lupulina* (black medic), *Medicago sativa* (alfalfa), *Melilotus albus* (white melilot), *Ononis spinosa* (spiny restharrow), *Pisum sativum* (pea), *Securigera varia* (crown vetch), and *Trifolium pratense* (red clover). Aphid collections took place in spring and summer 2011 in eastern and western France and consisted exclusively of wingless parthenogenetic females (Table 1). No specific permissions were required for these collection activities and samplings did not involve endangered or protected species. To prevent the detection in our sequencing analysis of bacterial taxa associated with internal parasites, field-collected aphids were grown individually on broad bean (the universal host for all pea aphid biotypes [23]) in controlled conditions ensuring clonal reproduction (16 hours light per day, 18°C). Parasitism rates ranged from 0% to 86% across pea aphid samples and were mainly due to parasitic wasps (e.g. *Aphidius ervi*) and fungal pathogens (e.g. *Pandora neoaphidis*). Healthy parthenogenetic females were let to reproduce for two generations and those that founded clonal lineages were kept alive for subsequent molecular analyses. Each clonal lineage was characterized at several microsatellite markers to assign genotype to biotype using a procedure detailed in [24]. Based on genotyping and clustering analyses, we then discarded all presumable copies of the same clone, the migrant individuals coming from other host races, and the hybrids between biotypes. Table 1 describes the different pea aphid samples considered in the present study.

**Table 1:** Host plant and geographic origins of pea aphid samples examined for intra-host bacterial diversity with 16S rDNA amplicon sequencing.

Host plant	Common name	Sample	Collection date	Site	GPS position
*Cytisus scoparius*	Common broom	Csco	August 2011	Lantenay	46°03'31'' N, 05°32'32'' E
*Lathyrus pratensis*	Meadow vetchling	Lpra	August 2011	Lantenay	46°03'31'' N, 05°32'32'' E
*Medicago lupulina*	Black medic	Mlup	August 2011	Lantenay	46°03'31'' N, 05°32'32'' E
*Medicago sativa*	Alfalfa	Msat-1	May 2011	Domagné	48°04'15'' N, 01°23'34'' W
*Medicago sativa*	Alfalfa	Msat-2	May 2011	Domagné	48°04'15'' N, 01°23'34'' W
*Melilotus albus*	White melilot	Malb	August 2011	Lantenay	46°03'31'' N, 05°32'32'' E
*Ononis spinosa*	Spiny restharrow	Ospi	August 2011	Lantenay	46°03'31'' N, 05°32'32'' E
*Pisum sativum*	Pea	Psat-1	May 2011	Domagné	48°04'15'' N, 01°23'34'' W
*Pisum sativum*	Pea	Psat-2	May 2011	La Chapelle Thouarault	48°07'27'' N, 01°51'58'' W
*Securigera varia*	Crown vetch	Svar	August 2011	Lantenay	46°03'31'' N, 05°32'32'' E
*Trifolium pratense*	Red clover	Tprat-1	May 2011	Domagné	48°04'15'' N, 01°23'34'' W
*Trifolium pratense*	Red clover	Tprat-2	May 2011	Domagné	48°04'15'' N, 01°23'34'' W

### DNA extraction and pyrosequencing of 16S rDNA amplicons

Adults of *A*. *pisum* from the distinct genotypes and biotypes were first surface-sterilized with 70% ethanol for 1 min, 10% bleach for 1 min and three washes of ultrapure water for 1 min. DNA was extracted from each individual (whole body) using the QIAGEN DNeasy kit following the manufacturer’s protocol. DNA extractions were then quantified using a Nanodrop spectrophotometer and DNA from 20 individuals (each with distinct genotypes but from the same biotype) were normalized and combined to create DNA pools. Twelve pools were constituted in total: two from alfalfa, clover and pea from each of the two sampled fields and one from the six other plant origins (Table 1). We used universal primers to amplify a V4-V5 variable region of the 16S ribosomal DNA (rDNA) in Eubacteria and Archebacteria that contains sequence variation to distinguish bacterial species. The 16S primers (F: 5’GTGCCAGCMGCCGCGGTAATAC 3’ and R: 5’ CCGTCAATTCCTTTGAGTTT 3’ from) are highly conserved in bacteria and amplify a region of about 420 bp. Additional sequences were added to the 5’ end of the primers for multiplexing of the samples and for Roche Titanium amplicon sequencing. Each fusion primer consisted of Adaptor A (5’ CCATCTCATCCCTGCGTGTCTCCGAC 3’) or Adaptor B (5’ CCTATCCCCTGTGTGCCTTGGCAGTC 3’) followed by a 4-mer key sequence (5’ TCAG 3’), a 5-mer to 10-mer Multiplex IDentifier (MID), and finally the 16S rDNA primer. In total, we used 12 different MIDs for both the forward and the reverse primers. The primers were HPLC purified and PCR amplification was performed in a volume of 25 μL with High Fidelity DNA polymerase (Roche). After initial denaturation for 2 min at 94°C, 33 cycles of denaturation for 30 sec at 94°C, annealing for 30 sec at 64°C and elongation at 72°C for 1 min were performed. For the last cycle, the elongation time was extended to 6 min. Polymerase Chain Reactions were duplicated for each pooled sample, each PCR product duplicate being then cleaned using the QIAquick PCR purification kit (Qiagen) following the manufacturer’s instructions and sequenced in both directions on half of a Roche 454 FLX Genome Sequencer plate using Titanium Series reagents at the Environmental Genomics platform of BioGenouest, University of Rennes 1, France.

### 16S rDNA sequence analysis

After application of filter criteria through manufacturer’s pipeline, sequencing on the GS FLX machine generated 425,410 sequences with a median size of 405 bp. Sequences were then processed using the workflow provided by MOTHUR [25]. During the demultiplexing step, all sequences with more than one mismatch with our MIDs were removed. To be conservative in our approach and to increase reliability in taxonomic assignment, we then kept only sequences with a length between 400 and 430 bp (expected size of the PCR product is about 420 bp). Finally, chimeric sequences that arise during PCR were eliminated using the UCHIME algorithm [26].

After these selection steps, sequences were aligned using the Greengenes Core Set alignment as a template [27]. The aligned sequences were then assigned to Operational Taxonomic Units (OTUs) based on the general approach implemented in MOTHUR which finds the closest Greengenes template for each read. OTUs that were present in only one of the two PCR replicates of each aphid sample were not considered in further analyses. Representative sequences of each OTU found in the different samples were then uploaded in a file that also contained reference 16S rDNA sequences of common bacterial symbionts of arthropods (Table 2). OTU and reference sequences were used to generate a Neighbor-Joining tree to assess the phylogenetic position of bacterial taxa present in pea aphid samples. The NJ tree was constructed using Clustal W version 2.0.11 [28] and visualized with MEGA 5 [29]. Genbank accession numbers of representative sequences of each OTU found in the different samples (74 sequences in total) and used to build the tree are given in S1 Table.

**Table 2:** Reference sequences of 16S rDNA genes from bacterial taxa associated with insect hosts (except *Spiroplasma* from *Citrus*).

Bacterial taxa	Host	Accession numbers
*Arsenophonus sp*.	*Stomaphis fagi*	gi|242397733|gb|FJ655540.1|
*Arsenophonus sp*.	*Bemisia tabaci*	gi|354508545|gb|JN204477.1|
*Buchnera aphidicola* (ref.1)	*Acyrthosiphon pisum*	gi|304030|gb|M27039.1|
*Buchnera aphidicola* (ref.2)	*Acyrthosiphon pisum*	gi|9588077|dbj|AB033774.1|
*Cardinium sp*.	*Bemisia tabaci*	gi|354508549|gb|JN204481.1|
*Cardinium sp*.	*Encarsia hispida*	gi|114054953|gb|DQ854695.1|
*Erwinia aphidicola* (ref.1)	*Acyrthosiphon pisum*	gi|340051242|emb|FN547376.1|
*Erwinia aphidicola* (ref.2)	*Acyrthosiphon pisum*	gi|359805495|dbj|AB681773.1|
*Erwinia aphidicola* (ref.3)	*Acyrthosiphon pisum*	gi|157073730|dbj|AB273744.1|
*Hamiltonella defensa*	*Acyrthosiphon pisum*	gi|56785712|gb|AY692361.1|
*Pantoea agglomerans*	*Acyrthosiphon pisum*	gi|57864392|dbj|AB004757.2|
*Pantoea agglomerans*	*Myzus persicae*	gi|57670121|gb|AY849936.1|
PAXS	*Acyrthosiphon pisum*	gi|226246999|gb|FJ821502.1|
*Regiella insecticola* (ref.1)	*Acyrthosiphon pisum*	gi|10567782|gb|AF293618.1|
*Regiella insecticola* (ref.2)	*Acyrthosiphon pisum*	gi|59709604|gb|AY907547.1|
*Rickettsia sp*. (ref.1)	*Acyrthosiphon pisum*	gi|70568364|dbj|AB196668.1|
*Rickettsia sp*. (ref.2)	*Acyrthosiphon pisum*	gi|1147763|gb|U42084.1|RPU42084
*Rickettsiella viridis* (ref.1)	*Acyrthosiphon pisum*	gi|315064938|dbj|AB522703.1|
*Rickettsiella viridis* (ref.2)	*Acyrthosiphon pisum*	gi|315064940|dbj|AB522705.1|
*Serratia symbiotica* (ref.1)	*Acyrthosiphon pisum*	gi|9588081|dbj|AB033778.1|
*Serratia symbiotica* (ref.2)	*Acyrthosiphon pisum*	gi|29569343|gb|AY136139.1|
*Spiroplasma sp*.	*Drosophila hydei*	gi|224797645|gb|FJ657213.1|
*Spiroplasma sp*.	*Harmonia axyridis*	gi|4582256|emb|AJ132412.1|
*Spiroplasma sp*.	*Acyrthosiphon pisum*	gi|13359308|dbj|AB048263.1|
*Spiroplasma citri* R8A2HP	*Citrus*	gi|310974985|ref|NR_036849.1|
*Spiroplasma syrphidicola*	*Eristalis arbustorum*	gi|219846121|ref|NR_025711.1|
*Wolbachia sp*.	*Cinara cedri*	gi|52355655|gb|AY620430.1|

### Bacterial community analyses

To analyze the bacterial community across the pea aphid samples, only presence-absence data were considered as both technological artifacts and innate biological traits bias relative quantification of each OTU abundance by 454 read counts [30]. The dissimilarity between the bacterial communities of pea aphid samples (beta diversity) was quantified by the Bray-Curtis distance, a metric based on the presence/absence of each OTU. Bray-Curtis dissimilarities between all pairwise combinations of pea aphid samples were summarized as a matrix (S2 Table). The Bray-Curtis dissimilarity matrix was ordinated following a non-metric multidimensional scaling (nMDS), a nonparametric ordination-based method where an iterative algorithm projects the multidimensional data of the dissimilarity matrix into a minimal dimensional space. The result of nMDS ordination is visualized on a scatter graph where the position of each pea aphid sample depends on its distance from all other points in the analysis. This method reduces ecological community data complexity and identifies meaningful relationships amongst communities. To analyze the effect of plant-based genetic differentiation in *A*. *pisum* on the bacterial community composition, we used a Mantel test to calculate the correlation between the matrix of Bray-Curtis dissimilarities between all pairwise combinations of pea aphid biotypes and the matrix of the genetic distance among these host-adapted aphid populations obtained from [31]. Finally, a Student test was performed to test whether the mean of the Bray-Curtis dissimilarities between biotypes is higher than the mean of Bray-Curtis dissimilarities within biotypes. The ‘vegan’ package available in R was used to calculate Bray-Curtis dissimilarities and to perform both the nMDS and the Mantel test.

## Results

### Overall bacterial diversity and taxonomic assignments

**Fig 1:**
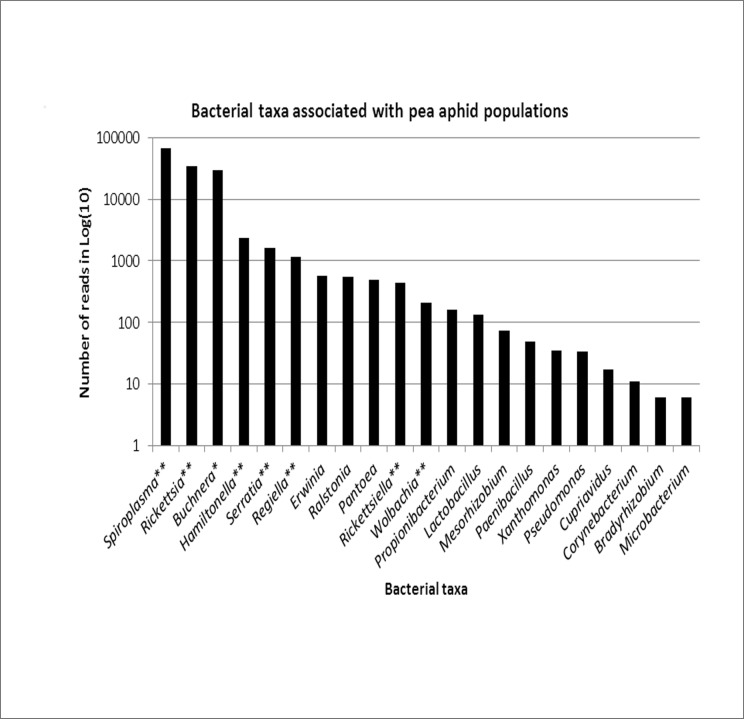
Number of 16S rDNA reads in Log(10) for the 21 bacterial taxa detected in nine plant-specialized biotypes of the pea aphid, *Acyrthosiphon pisum*. *obligatory symbiont and **facultative symbionts.

Following our selection steps on sequence quality, size and redundancy between PCR replicates, 138,539 sequences were obtained, with a mean of 11,545 reads per sample. Overall, these sequence reads were classified into 21 Operational Taxonomic Units (OTUs). However, only 13 OTUs had a frequency exceeding 0.1% (i.e. approx. 140 reads) among which 3 OTUs represented more than 94% of the sequences. The classification from Greengenes database resulted in 21 unique bacterial genera that belonged to 4 phyla, 6 classes, 8 orders and 14 families (S3 Table). The Proteobacteria represented 51% of these classified bacteria, with the Alphaproteobacteria and the Gammaproteobacteria being the commonest taxa with 25% and 26%, respectively. Among the 138,539 sequences of 400–430 bp, 98% were assigned to bacterial taxa already reported as heritable symbionts of aphids. *Spiroplasma* was the most represented taxon in number of sequences (48%) followed by *Rickettsia* (25%) and *Buchnera* (21%). The frequencies of the other pea aphid symbionts were <2% (Fig 1). We confirmed the presence of *Wolbachia*, which was recently reported in North American *A*. *pisum* by [14], in two pea aphid samples (total frequency of 0.15%). Apart from these symbionts, 454 sequencing detected other bacteria not known to be heritable in arthropods, although most did not exceed 0.1% of sequence reads. Among those with frequencies above 0.1% were *Erwinia*, *Pantoea*, *Propionibacterium* and *Ralstonia*, each being found in multiple populations and for a total of more than 150 reads each. *Erwinia* and *Pantoea* have been described as aphid gut associates [32, 33]. *Ralstonia* encompass several soil borne plant pathogens that could be ingested or vectorized by aphids. *Propionibacterium* are usually found in the skin of humans and other animals and their occurrence in our 454 reads may represent contaminants from human handling. Bacterial taxa with read abundance below 0.1% were either plant associates such as *Rhizobium* and *Xanthomonas* or environmental/contaminant bacteria.

**Fig 2:**
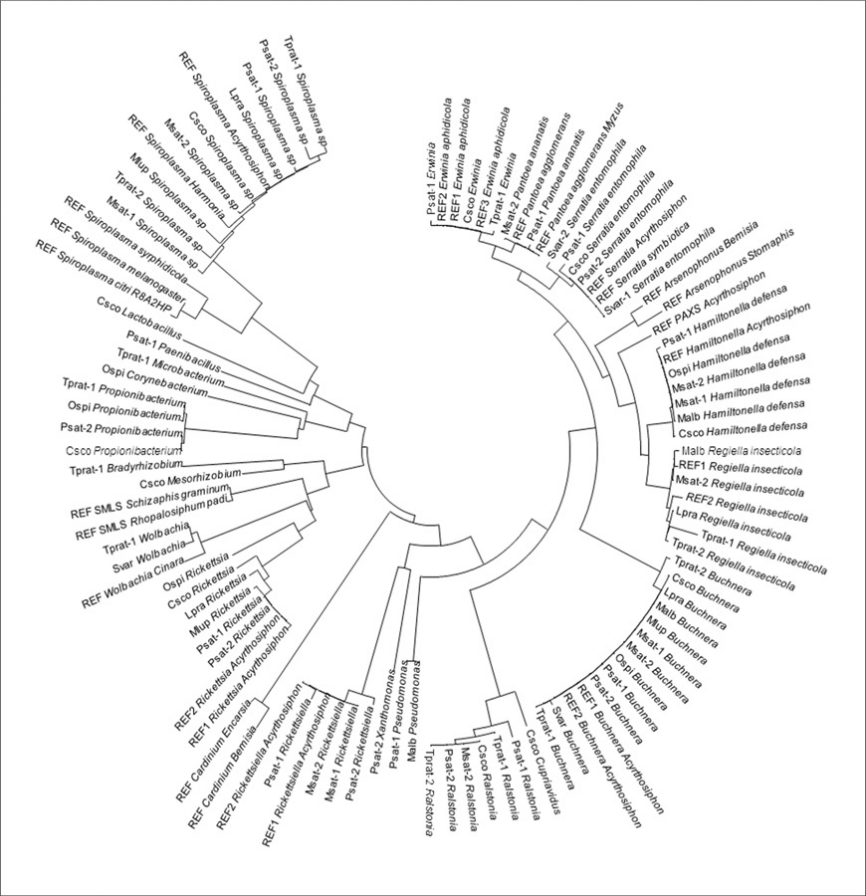
Phylogenetic analysis of 16S rDNA based on a Neighbor-Joining tree relating bacterial sequences from references selected in GenBank (REF) and from 454 amplicon sequencing of populations and biotypes of the pea aphid complex. Accession numbers of representative sequences of each OTU found in the different samples (74 sequences in total) are given in Supporting Information (S3 Table).

Taxonomic assignment based on the Greengenes database was very well supported by the Neighbor-Joining tree of 16S rDNA sequences from our 454 dataset and from references selected in GenBank (Fig 2). OTUs assigned to reported aphid symbionts clustered with the reference sequence from a pea aphid host. None of our 454 sequences grouped with the PAXS reference, probably because this recently described pea aphid symbiont was not detected due to poor sequence identity with our universal primers (sequence homology with PAXS was 100% for the reverse primer but only 60% for the forward primer). *Erwinia* and *Pantoea* appear as two distinct clades on the phylogenetic tree. Globally, there was a significant range of 16S rDNA variation within bacterial taxa, which could be due to either sequence errors in both 454 and Genbank sequences or variation between strains of bacterial taxa from different populations or biotypes of *A*. *pisum*.

### Bacterial community composition and host-adapted pea aphid populations

**Fig 3:**
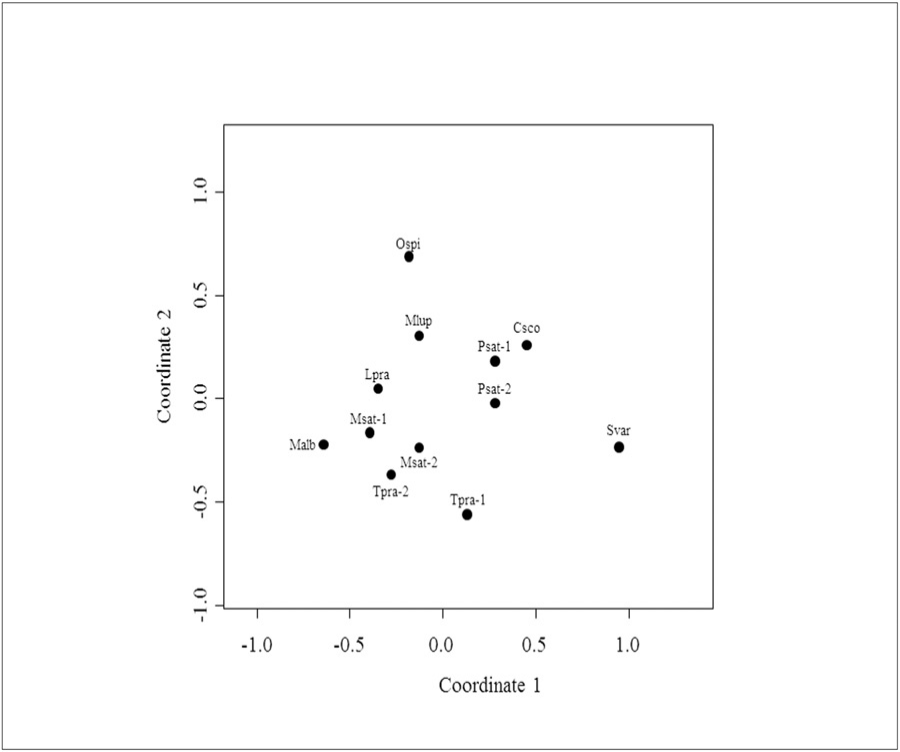
Non-metric MDS ordination plot comparing bacterial communities from different pea aphid samples. Each data point represents the bacterial community identified from a single sample (see Table 1 for dot label legends). The Bray-Curtis dissimilarity index was used to rank distances calculated using the presence-absence community data. Stress of the nMDS = 0.152.

**Fig 4:**
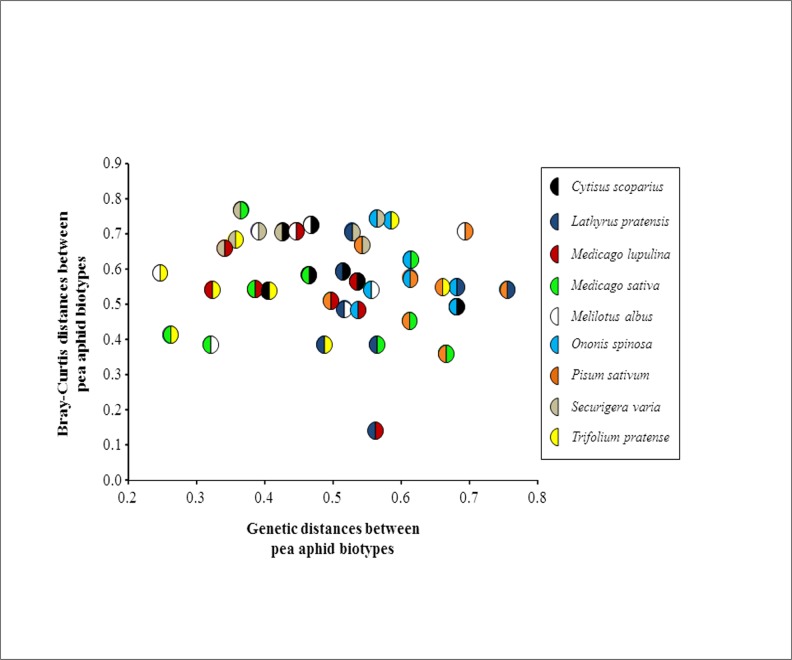
Relationship between the matrix of Bray-Curtis dissimilarities between all pairwise combinations of pea aphid biotypes and the matrix of the genetic distance among these host-adapted aphid populations. Each data point represents a pairwise combination of pea aphid biotype (Mantel test, p>0.05).

Bacterial diversity in each pea aphid sample (alpha diversity) was relatively low with 3–11 unique OTUs per sample (mean = 6.2). Several bacterial OTU were shared across pea aphid samples but some samples were characterized by specific bacterial communities (Table 3). Not only were the bacterial communities of pea aphid samples dominated by only a few OTUs (low alpha diversity), but the distribution of the bacterial OTUs was often limited to only a few pea aphid samples (high beta diversity). Bray-Curtis dissimilarity indices calculated on the 21 validated OTUs distributed among our samples revealed that populations from the same pea aphid biotype harbor similar bacterial communities (Fig 3). The statistical analysis confirmed this pattern since the bacterial community dissimilarity was significantly lower within than among pea aphid biotypes (respectively 0.31±0.07 and 0.55±0.02, t = 3.56, p = 0.028). Finally, the difference of bacterial community composition across the pea aphid biotypes was not related to the phylogenetic distance between these host-adapted aphid populations (Mantel test, r = -0.102, p = 0.634, Fig 4).

**Table 3:** Distribution of the 21 bacterial OTU across samples from various populations and biotypes of the pea aphid.

OTU type	OTU name	Pea aphid samples												OTU occurrence
		Csco	Lpra	Mlup	Msat-1	Msat-2	Malb	Ospi	Psat-1	Psat-2	Svar	Tpra-1	Tpra-2	
**Aphid symbionts**	*Buchnera*	X	X	X	X	X	X	X	X	X	X	X	X	12
	*Hamiltonella*	X			X	X	X	X		X				6
	*Regiella*		X		X	X	X					X	X	6
	*Rickettsia*	X	X	X				X	X	X				6
	*Rickettsiella*				X	X			X	X				4
	*Serratia*	X							X	X	X			4
	*Spiroplasma*	X	X	X	X	X			X	X		X	X	9
	*Wolbachia*										X	X		2
**Gut associates**	*Erwinia*	X								X		X		3
	*Pantoea*					X				X				2
**Plant associates**	*Bradyrhizobium*											X		1
	*Mesorhizobium*	X												1
	*Pseudomonas*						X			X				2
	*Ralstonia*	X				X			X	X		X	X	6
	*Xanthomonas*								X					1
**Environmental bacteria**	*Cupriavidus*	X												1
	*Corynebacterium*							X						1
	*Lactobacillus*	X												1
	*Microbacterium*											X		1
	*Paenibacillus*									X				1
	*Propionibacterium*	X						X	X			X		4
**OTU number**		11	4	3	5	7	4	5	8	11	3	9	4	

Names of samples are those indicated in Table 1.

## Discussion

Analyzing the microbiome of several pea aphid biotypes using deep 454 pyrosequencing of 16S rDNA genes reveals a relatively low bacterial diversity, with a total of 21 valid taxa detected and about 6 OTUs per sample on average. This study also shows that the pea aphid microbiota is dominated by very few symbionts, with 98% of the 138,539 reads assigned to the eight bacterial taxa already described as symbionts of this species.

Before discussing further these key results, caution should be taken with data generated by sequencing of 16S rDNA gene amplicons as this method suffers from several limitations [34]. First, the choice of universal primers may bias amplification towards taxa with higher sequence affinity, and sequence abundance per OTU may not reflect bacterial abundance [30]. Because of these possible biases, we decided to analyze qualitatively and not quantitatively our sequence data by taking into account only the presence or absence of OTUs among samples. Here, these artifacts could explain why *Spiroplasma*, and not the obligatory symbiont *Buchnera* as one would expect, showed the highest abundance of 454 reads in our dataset. Second, the sequencing depth may limit the detection of rare taxa, leading to an underestimation of microbial diversity. However, 11,500 reads per sample were obtained on average here, a sequencing effort which is likely to capture most if not all of bacterial diversity and indeed rarefaction curves reached saturation in all samples (data not shown). Third, the size and quality of sequences may also prevent the accurate taxonomic assignment of OTUs formed by the clustering analysis of 16S rDNA sequences. Here, we applied stringent criteria to filter the 454 reads, by removing two third of them and keeping only high quality and long (400–430bp) sequences. Phylogenetic analysis of 16S rDNA sequences generated by 454 amplicon pyrosequencing validated taxonomic assignment since sequences assigned to bacterial taxa associated with aphids clustered with the reference sequences.

With these cautions in mind, our deep sequencing of 16S rDNA amplicons from multiple populations and biotypes of the pea aphid complex reveals the limited diversity of aphid bacterial communities, which are dominated by symbionts among which facultative symbionts vary drastically between host-specialized biotypes and less between localities. This strong link between plant specialization and bacterial communities confirms previous studies in European and American populations of *A*. *pisum* based on specific PCR-based detection of a range of facultative symbionts [20], [23], [35], [36], [37]. Such a relationship between plant specialization and microbial communities has been described in other insect species, including aphids [6], [38], [39], [40], [41]. However, it is uncertain whether symbiont communities associated with herbivores exert a direct influence on plant specialization. In the pea aphid complex, host ecology more than geography seems to shape symbiont composition [20] but we lack firm conclusions about the direct involvement of facultative symbionts in plant adaptation of their aphid hosts. It has been shown that *Regiella*-infected pea aphids present increased performances on white clover compared to those deprived of this facultative symbiont [16]. However, other studies have contradicted this finding [42], [43] and generalization to other symbionts does not support symbiont-driven plant specialization in *A*. *pisum* [4], [44]. Since the genetic divergence between pea aphid biotypes is not related with their difference in microbial communities (Fig 4), historical factors seem to have no or very little role in shaping symbiont composition in the complex. Therefore, ecological forces other than the direct plant influence (e.g. enemy pressure), as well as horizontal transfers are likely to be key factors in driving symbiont community structure among host-adapted biotypes [20], [44], [45].

Here, we detected *Wolbachia* in two biotypes (*Securigera* and *Trifolium*-adapted biotypes), confirming the recent discovery of this endosymbiont in *A*. *pisum* in North America and China [14], [46]. However, the prevalence of this symbiont, and therefore its impact on host fitness, must be very limited since it has been searched intensively before its recent detection [47], [48] and was found in only one *A*. *pisum* individual from North American populations among 318 screened [14]. Larger screenings on more biotypes and individuals are needed to better assess its distribution and prevalence across the pea aphid complex. More work is also required to examine the localization of *Wolbachia* in the insect, its potential effects and their nature on the host’s phenotype and fitness [14], [46], [47].

Besides heritable symbionts known for the pea aphid, *Erwinia* and *Pantoea* were detected in four samples with a substantial number of reads (>0.1%, 1068 reads in total). Interestingly these two bacteria are ubiquitous plant pathogens but have been also described as gut associates in *A*. *pisum* as well as in other aphid species [49], [33], [39], [50]. It is not clear whether these two gut bacteria are aphid pathogens, commensals, mutualists or use aphids as alternative hosts to plants [51], [52], [53]. *Erwinia aphidicola*, which has been isolated from the pea aphid gut and described by [32, 33] shows virtually complete 16S rDNA sequence homology with the three representative sequences of the OTU assigned to *Erwinia* in our dataset. *E*. *aphidicola* has been reported as pathogen for the pea aphid although negative effects have been detected in laboratory conditions and with concentration that may not be realistic [53]. It may be that *E*. *aphidicola* provides some benefit to the aphid under certain ecological conditions as shown for *Erwinia* bacteria which are gut associates of thrips and have diet-dependent effects on their insect hosts [54]. *Pantoea agglomerans*-like bacteria have been identified in phylloxera-inducing species where its prevalence can reach 100% as in some *Daktulosphaira vitifoliae* populations [39], [50]. Sequences of the OTU assigned to *Pantoea* clustered into the same clade as the reference sequences for *P*. *agglomerans*, suggesting that some pea aphid individuals are infected by this taxon which is related to phytopathogenic bacteria, but can apparently colonize insect gut with totally unknown effects. It is unlikely that these two gut bacteria are transmitted vertically because their chances of transmission from mother to offspring are small [49], [55]. It is more plausible that aphids acquire these bacteria either from the honeydew where they are probably excreted or from the plant surface [56], [57]. Another possibility is that they are ingested from plant sap, providing that these bacteria may circulate into the phloem [58]. Further investigations are required to ascertain the importance of aphid gut associates in both laboratory and field conditions.

The remaining bacterial taxa detected in samples of pea aphid biotypes and populations had low read abundance and were either plant associates such as *Rhizobium* and *Xanthomonas* or environmental/contaminant bacteria. Although their effect on aphids either as hosts or vectors of plant pathogens may not be negligible [52], their presence requires confirmation through a larger survey.

In this study, host-specialized biotypes that were collected from the field were then grown on broad bean, the universal host-plant for all biotypes, during two generations. This step, while reducing the background noise of microbes associated with internal parasites, might have provoked changes in the microbiota associated with the host, in particular in the diversity of non-heritable bacteria. Comparison of microbiota of aphids directly taken from the field with present results is needed to assess quantitative and qualitative differences in microbial communities between field and laboratory conditions. In addition, shift in host-plant (from native plant to universal host) imposed by our experimental design might have altered the microbial community associated with pea aphid biotypes and populations. However, this hypothesis is unlikely, at least for heritable symbionts whose vertical transmission is almost perfect and loss, very uncommon [3].

In conclusion, despite the artifacts inherent to sequencing of 16S rDNA gene amplicons and possible changes in microbial diversity resulting from aphid culture under laboratory conditions, our study provides qualitative results consistent with previous works based on taxon-specific PCR-based detection and more recent ones using without *a priori* approaches. In line with these studies, we found that bacterial communities associated with pea aphid hosts are largely dominated by a few symbiont taxa whose distribution varies drastically among biotypes. Among herbivore insects examined so far, sap-feeders such as aphids, psyllids and whiteflies show the poorest microbial diversity, with less than 12 OTUs per sample [14], [34], [59], [present study], which has been attributed to several not mutually exclusive factors involving nutrition, immunity and antagonisms [34]. While this diversity may be enriched by transient bacterial taxa (e.g. on the cuticle or in the gut) or by other microbes such as viruses and fungi, the ecological and evolutionary importance of these additional microbial associates for their hosts would probably be limited compared to obligate and facultative symbionts.

Our work leaves unresolved several important issues regarding the impact of microbial communities on their aphid hosts at individual, population and species levels. These include notably the effective importance and role of gut associates as well as the nature and relative influence of forces that shape microbial community structure, with particular attention to ecological factors and transmission patterns. Accessing the range and distribution of microbial diversity with NGS technologies applied to host populations from a large array of environmental conditions will certainly help in resolving these key issues, completed by more focused biological and functional analyses.

## Supporting Information

S1 Table Genbank accession numbers of 16S rDNA sequences of each OTU found in the different samples of pea aphids and assigned to bacterial taxa. (XLSX)

S2 Table Matrix of Bray-Curtis dissimilarities between all pairwise combinations of pea aphid samples. (XLSX)

S3 Table Taxonomic affiliation of the 21 bacterial OTUs detected in nine plant-specialized biotypes of the pea aphid, *Acyrthosiphon pisum*. Numbers and percentages of 16S rDNA reads are given for each taxon.
(XLSX)
